# Priority Setting for Pandemic Influenza: An Analysis of National Preparedness Plans

**DOI:** 10.1371/journal.pmed.0030436

**Published:** 2006-10-17

**Authors:** Lori Uscher-Pines, Saad B Omer, Daniel J Barnett, Thomas A Burke, Ran D Balicer

## Abstract

The authors provide a targeted review of national pandemic influenza plans from the developed and developing world, describing national variations in prioritization of vaccines and antiviral medications.

Recognizing the potential severe impact of pandemic influenza, the World Health Organization (WHO) urges every country to develop or maintain an up-to-date national influenza preparedness plan, and provides guidance on plan content [1]. WHO guidance focuses on numerous issues, from surveillance and communications to prioritization of vaccine. Despite the perceived imminence of this threat, WHO estimated that as of December 2005, only 40 countries had developed written plans [2]. Among these plans, there was a great deal of variation with respect to comprehensiveness, quality, and stage of completion [3].

## Prioritizing Scarce Resources

Prioritization of scarce pharmaceutical resources (e.g., vaccines and antiviral medications) that could ultimately delay the spread of a pandemic or lower overall incidence is of particular importance to planning at the national level. Because of costs and manufacturing limitations, these critical resources are likely to be scarce and will require evidence-based rationing. At current capacity, we cannot expect to vaccinate more than 14% of the world's population within a year of a pandemic [4]. Similarly, although manufacturing capacity has recently quadrupled, it is estimated to take a decade to produce enough of the antiviral medication oseltamivir for 20% of the world's population [3]. Because estimates of global demand for these resources depend on the priorities of individual countries, priority setting at the national level is the first step toward global preparedness [5].

Until recently, developing countries were unlikely to secure supplies of oseltamivir. Today, generic drug manufacturers in Bangladesh, Algeria, India, and China produce oseltamivir, some for local use as determined in official agreements and some for use in areas without patent protection [6]. It seems that the world is entering a phase in which pharmaceutical interventions will not be limited to the developed world—e.g., by spring of 2006, 65 countries had ordered stocks of oseltamivir [7]. Thus, prioritization is crucial for all nations.

Despite the growing availability of pharmaceutical resources, the planning community has yet to conduct a critical analysis of distribution strategies. A study from April 2006 that reviewed national pandemic influenza plans briefly touched on prioritization among a myriad of competing issues. Furthermore, it focused exclusively on the European region, reviewing a total of 21 plans [8]. Given the integral role of priority setting in the concept of preparedness and the ethical, political, and public health implications of deciding who receives potentially life-saving interventions, we sought to perform a targeted review of national pandemic influenza plans from developed and developing countries. We aim to describe the global variation in national-level prioritization of vaccines and antiviral medications, and to inform future priority-setting processes. Our search strategy is shown in Box 1.

## National Preparedness Plans

### Sample description.

We obtained a total of 50 pandemic influenza plans. We identified these plans through Web sites that compile lists of plans (39 plans), contacts at national influenza centers (five plans), Google searches (five plans), and personal contacts (one plan). We subsequently excluded three plans (Finland, Italy, and Sweden) that were not available in the selected languages, and two plans (Sierra Leone and Namibia) that focused exclusively on avian influenza preparedness. Our final sample contained 45 national pandemic influenza plans, 19 from developed and 26 from developing nations (Box 2).

Three plans were from low-income nations, a World Bank–defined category with 59 total members. The subset sample (see Box 1 for a description of this sample) contained 25 English-language plans that prioritized at least one pharmaceutical intervention. In total, the plans represented about 3.8 billion individuals, or roughly two-thirds of the world population.

**Box 1. Search Strategy for Identifying National Pandemic Influenza Plans**

We searched for pandemic influenza plans on official Web sites that compile planning documents (e.g., http://www.who.int and http://www.undg.org). We used Google to identify links to documents on ministry of health Web sites—search keywords included “pandemic,” “influenza” (as well as synonyms and foreign-language translations), “plan,” and names of countries. Lastly, we contacted the WHO National Influenza Centers for 83 countries, and requested access to official pandemic influenza plans. The search process occurred between February 6, 2006, and April 20, 2006.

For the study, we included all plans that addressed human impact of pandemic influenza, regardless of whether it was available in draft or final form, published or unpublished. Furthermore, the official plans of any self-governing territory—regardless of nationhood—were included. For the purpose of this Policy Forum, the term “nation” refers to both recognized countries and territories. We excluded documents that pertained solely to avian influenza preparedness and response. We also excluded plans that were not available in English, Spanish, French, or German.

We designed a data abstraction form to guide a systematic exploration of 44 plan variables. We selected variables with reference to WHO guidelines: vaccine and antiviral priority groups, rankings of groups, goals of pharmaceutical interventions, and the inclusion of modeled scenarios. For a subset of English-language plans that prioritized at least one pharmaceutical intervention, we collected data on rationales for prioritization, definitions of “high-risk” and “essential services” groups, and references to ethical considerations. In addition to extracting data from planning documents, we gathered national-level data on demographics (e.g., population size) from World Bank sources [15]. We classified level of development according to the World Bank criteria, defining low- and medium-income nations as “developing” and high-income nations as “developed” [16]. Of the 45 plans that met inclusion criteria, 11 were not available in English translation. In these cases, the research team worked with experienced translators, who orally translated relevant sections. We used STATA statistical software (College Station, Texas) and Microsoft Excel (Redmond, Washington) for data management and analysis.

The subset sample included one plan from Africa, one from Australia, three from North America, seven from Central and South America, 16 from Europe, and 17 from Asia–Pacific. As of April 20, 2006, 18 (40%) nations had identified cases of H5N1 in bird populations [9]. The date of pandemic influenza plan release, or publication, ranged from October 1997 (Japan) to January 2006 (Israel, Argentina, France, and Venezuela). There were 28 (62%) plans dated after June 2005. Plans ranged in length from nine pages to 453 pages, with most in the 25–60 page range.

### Prioritization description.

Goals of pharmaceutical interventions were recorded for 21 plans. Although the wording varied slightly, three goals appeared in various combinations. These goals were reduction of morbidity and mortality (21 plans), continued maintenance of essential services (13 plans), and minimization of social and economic impacts (13 plans). Within their plans, 18 (40%) nations—15 developed and three developing—included a modeled scenario to estimate pandemic impact in terms of morbidity and mortality. Estimated attack rates ranged from 10% (Ireland, Thailand, and the United Kingdom) to 50% (Greece and the UK), with seven nations modeling several different attack-rate scenarios. Stated mortality rates ranged from 0.37% to 2.5%, and four nations specifically based assumptions on worst-case scenarios derived from the 1918 pandemic.

There were 14 (74%) developed and 14 (54%) developing nations that prioritized population groups to receive vaccine in a pandemic (p = 0.18), and 12 (63%) developed and 10 (38%) developing nations that prioritized population groups to receive antivirals (p = 0.10) (Table 1). No national plans included prioritization schemes for the distribution of nonpharmaceutical medical resources, such as ventilators or N95 masks.

**Table 1:**
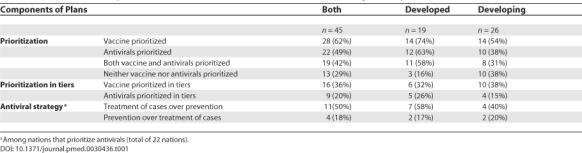
Prioritization Components of Pandemic Influenza Plans: All Countries and by Development Status

Of the 28 nations that prioritized vaccine, high-risk individuals were prioritized most frequently, followed by health-care workers and essential services workers—a category which may include health-care workers depending on a nation's definition (Figure 1). Certain nations listed health-care workers independently, or alternatively chose to have the category of essential services represent a wide rage of workers including health-care personnel. Some nations grouped children and the elderly under the catch-all category of high-risk individuals. In at least two plans, 18 distinct high-risk groups appeared. For antiviral distribution, the major groups remained the same, but had a different ordering of health-care workers, essential services workers, and high-risk individuals (Figure 2). Among the nations that prioritized vaccine, 13 (47%) included children in their schemes and 11 (39%) included key decision makers.

**Figure 1:**
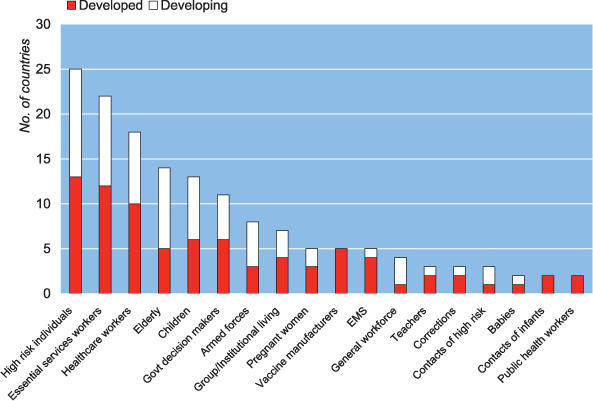
Vaccine Priority Groups by Development Status—Listed in at Least Two National Plans

**Figure 2:**
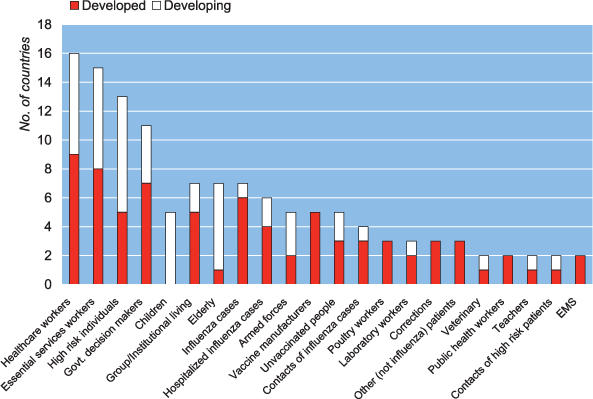
Antiviral Priority Groups by Development Status—Listed in at Least Two National Plans

Three options guided the antiviral distribution strategies detailed in 22 plans: pre-exposure prophylaxis, postexposure prophylaxis, and treatment of influenza cases within 48 hours of onset of illness. Seven (32%) nations did not differentiate among these strategies in allocating resources. Eleven (50%) plans indicated that the treatment of cases would be preferred over prophylaxis for groups such as health-care workers. Among the four nations that allocated antivirals for prophylaxis over treatment, two were from the developing world. The UK plan, which prioritized treatment over prophylaxis, reasoned that long-term prevention at the population level was not an efficient use of resources. Serbia's plan prioritized prophylaxis because the 48-hour time window required for effective treatment was considered too narrow.

Health-care workers were consistently ranked at the top of the vaccine and antiviral priority lists (Table 2). Eleven nations (73% of nations that ranked) prioritized health-care workers as Tier 1 for vaccine distribution, and six nations (67%) prioritized this group as Tier 1 for antiviral distribution. After Tier 1, there was greater variability in groups that fell into the subsequent tiers. For vaccine Tier 2, six nations (40%) prioritized essential services workers. For antiviral Tier 2, three nations (33%) prioritized health-care workers, and two (22%) prioritized essential services workers. Seven (44%) nations used the following tiered vaccine scheme: (1) health-care workers, (2) essential services workers, and (3) high-risk patients. All other orderings were unique.

**Table 2:**
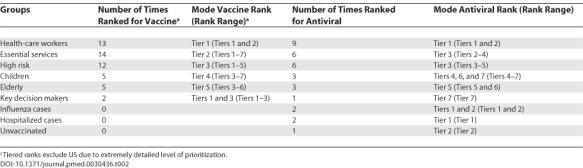
Selected Vaccine and Antiviral Prioritization Groups

Of the 25 subset plans, 14 described the composition of the high-risk category (Table 3). Twelve plans included immunocompromised individuals in this category. The other most common members of the high-risk category were individuals with cardiovascular disease (11 plans) and the elderly (10 plans). The composition of the essential services group was also described in plans. The most common members of this group were communications/telecommunications workers (15 plans), fire fighters (14 plans), and energy/power supply workers (14 plans.)

**Table 3:**
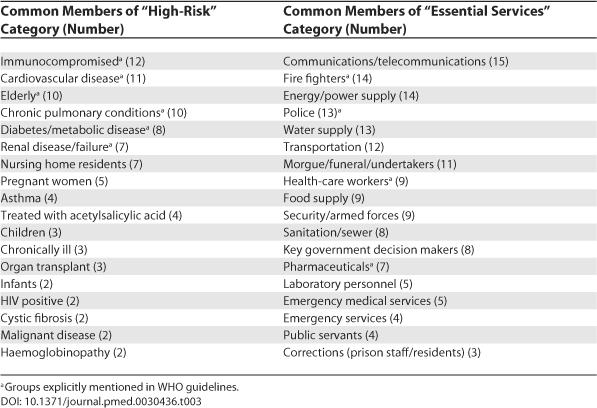
List and Frequency of Common Members of “High Risk” and “Essential Services” Categories in 25 Plans (Prioritized by At Least Two Nations)

### Rationales for prioritization.

Of 25 plans, 13 (52%) provided rationales for the inclusion of certain priority groups. Two plans explained the exclusion of certain groups (e.g., children and families of health-care workers). Plans justified the prioritization of health-care workers for the following reasons: (1) they are at increased risk of acquiring infection and/or passing it to vulnerable patients (Australia, Bahrain, Czech Republic, Israel, the UK, and the United States); (2) they perform essential services, enable the treatment of those affected by disease, and are required for quality medical care and good patient outcomes (Australia, Bahrain, Canada, Ireland, Israel, Serbia, Montenegro, the UK, and the US); and (3) their availability reduces morbidity and mortality (Australia, Bahrain, Canada, and Ireland).

Furthermore, plans justified the inclusion of children for the following reasons: (1) since children transmit influenza, prioritizing this group is consistent with the goal of containment (Australia, Bahrain, and Norway), and (2) children may have a great risk of infection and severe illness depending on the circulating strain (Brazil and Thailand). Canada's plan explained its exclusion of children, stating that this group is at the lowest risk of developing severe outcomes from influenza during annual epidemics, and absence from school does not have the direct economic impact that illness in adults has. The US plan excluded children because in prior pandemics and epidemics, this group has been at low risk for hospitalization and death.

## Discussion

This study provides the first description of pandemic influenza prioritization decisions in both the developed and the developing world. Although WHO recommends that nations set priority groups for both vaccine and antiviral distribution [5], about 30% prioritized neither resource.

Fewer nations prioritized antivirals (49%) than vaccine (62%). This is an unexpected finding since antivirals may be the first—and, perhaps, the only—pharmaceutical intervention available to many countries in a pandemic [10]. Because it is estimated to take six months to mass produce strain-specific vaccine, and global antiviral production and stockpiling is increasing, priority setting for antivirals may prove to be more critical to pandemic preparedness. It is becoming increasingly essential for nations to attend to both allocation schemes in their plans, and the planning community can support this effort by emphasizing the relative importance of antiviral medications.

The nations that prioritized vaccines and antivirals varied considerably in their allocation decisions, and in several instances, chose to deviate from WHO guidelines. This variation cannot be explained with respect to stated goals; rather, we must look to differences in interpretation of evidence or sociocultural factors. It seems nations were equally divided with respect to children, with 47% prioritizing children for vaccine against WHO recommendations. WHO states, “There is no evidence that the use of inactivated vaccine in children will reduce the spread of a pandemic in the community, and this strategy is not recommended” [5]. The decision to prioritize children (although in a rather low rank, Tiers 3–7) may reflect differing interpretations of evidence. While evidence from clinical trials and observational studies remain unclear, two recent modeling studies indicated that vaccinating children may reduce influenza transmission in the community [11,12].

Since these models were not available when most of the plans were written, the decision to prioritize children may also reflect different sociocultural values, suggesting that purely epidemiology-based guidelines are insufficient in priority setting. Planners can specifically research and consider alternatives to preferential vaccination that both recognize the moral imperative to prioritize children and achieve optimal public health impact. (The academic literature has begun to discuss this ethical question. A recent article by Emanuel and Wertheimer argued in favor of prioritizing individuals between early adolescence and middle age, contrary to existing recommendations [13].)

**Box 2. Pandemic Influenza Plans: Country, Date of Publication/Release, and Web Site**

**Argentina** (2006)^a^

**Australia** (June 2005) http://www.health.gov.au/internet/wcms/publishing.nsf/Content/ohp-pandemic-ahmppi.htm

**Austria** (September 2005) http://www.bmgf.gv.at/cms/site/attachments/3/6/8/CH0019/CMS1126084167391/pandemieplanh3neu.pdf

**Bahrain** (November 2005) http://ai.moh.gov.bh/BahrainPlan.asp

**Bolivia** (October 2005)^a^

**Brazil** (September 2005) http://portal.saude.gov.br/portal/arquivos/pdf/plano_flu_english.pdf

**Canada** (September 2004) http://www.phac-aspc.gc.ca/cpip-pclcpi/index.html

**Chile** (September 2005) http://www.minsal.cl/ici/pandemiainfluenza/ pandemiainfluenza.html

**China** (Unlisted) http://www.undg.org/content.cfm?id=1598

**Cuba** (October 2005) http://www.undg.org/content.cfm?id=1552

**Czech Republic** (April 2004) http://www.who.int/csr/disease/influenza/czechplan.pdf

**Fiji** (November 2005)^a^

**France** (January 2006) http://www.grippeaviaire.gouv.fr/IMG/pdf/Plan_version_anglaise.pdf

**Germany** (July 2005) http://www.rki.de/cln_006/nn_225576/DE/Content/InfAZ/I/Influenza/influenza__node.html__nnn=true

**Greece** (October 2005) http://www.who.int/csr/disease/influenza/nationalgreece.pdf

**Hong Kong** (February 2005) http://www.chp.gov.hk/files/pdf/flu_plan_framework_en_20050222.pdf

**Hungary** (September 2003) http://www.who.int/csr/disease/influenza/Pandemic_preparedness_plan_Hungary.pdf

**India** (December 2005) http://www.undg.org/content.cfm?id=1684

**Ireland** (2002) http://www.doh.ie/pdfdocs/panflu.pdf

**Israel** (January 2006)^a^

**Japan** (October 1997) http://www.mhlw.go.jp/english/topics/influenza/index.html

**Lithuania** (Sept 2005)^a^ http://www.vvspt.lt/aktai/gripas/2005%2009%2020%20GRIPO%20PLANO%20VERT.doc

**Mexico** (Unlisted) http://www.dgepi.salud.gob.mx/pandemia/planpp.htm

**Montenegro** (October 2005) http://www.mzdravlja.cg.yu/vijesti.php?akcija=rubrika&rubrika=188

**Nauru** (Aug 2005) http://www.spc.int/phs/PPHSN/Outbreak/Influenza/Pand-Preparedness-plans-Pacific-countries.htm

**New Caldonia** (October 2005) http://www.dass.gouv.nc/static/infoPratique/documents/PandemieGrippePlanActionNouvelleCaledonie20042005version131005.PDF

**New Zealand** (November 2005) http://www.moh.govt.nz/moh.nsf/ea6005dc347e7bd44c2566a40079ae6f/5f5694e4a5736dd2cc256c55000788a3?OpenDocument

**Norway** (July 2003) http://www.dep.no/archive/hdvedlegg/01/08/Influ041.pdf

**Palau** (October 2005) http://www.spc.int/phs/PPHSN/Outbreak/Influenza/Palau_Flu_Plan_Final_Draft_103105.pdf

**Philippines** (October 2005) http://www.undg.org/content.cfm?id=1671

**Poland** (2005) http://www.gis.gov.pl/index.php?option=com_content&task=category&sectionid=10&id=28&Itemid=61&lang=iso-8859-2

**Serbia** (October 2005) http://www.zdravlje.sr.gov.yu/downloads/pdf_e/Influenza%20Preparedness%20Plan.pdf

**Singapore** (December 2005) http://www.moh.gov.sg/corp/hottopics/influenza/detail.do

**Slovak Republic** (August 2001) http://www.who.int/csr/disease/influenza/Pand_Plan_SR_AJ.pdf

**South Africa** (Unlisted) http://www.undg.org/content.cfm?id=1528

**Spain** (May 2005) http://www.msc.es/ciudadanos/enfLesiones/enfTransmisibles/docs/PlanGripeIngles.pdf

**Switzerland** (March 2005) http://www.bag.admin.ch/influenza/01120/01134/index.html

**Thailand** (May 2005) http://thaigcd.ddc.moph.go.th/AI_Nationalplan_en_05_07.html

**Timor Leste** (November 2005) http://www.undg.org/content.cfm?id=1551

**United Kingdom** (October 2005) http://www.dh.gov.uk/PolicyAndGuidance/EmergencyPlanning/PandemicFlu/fs/en

**Uruguay** (Unlisted) http://www.undg.org/content.cfm?id=1572

**United States** (November 2005) http://www.hhs.gov/pandemicflu/plan

**Venezuela** (January 2006) http://www.msds.gov.ve/msds/modules.php?name=Content&pa=showpage&pid=422

**West Bank/Gaza** (2005) http://www.healthinforum.net/files/misc/nat_avian_plan.pdf

**Yemen** (October 2005) http://www.undg.org/content.cfm?id=1608

It is notable that despite the influence of sociocultural factors on decisions, priority-setting rationales always referred to epidemiology-based arguments. None of the plans prioritized children as a special group in need of protection, and plans seldom mentioned ethics in the context of resource allocation. Within the 25 plan subset, only five referred to concepts of equity and fairness in allocation decisions, and two referred to consultation with ethicists.

In the absence of an explicit WHO guideline, more than half the nations that prioritized vaccine distribution developed a tiered (ranked) strategy, revealing another example of variation. It may seem that the more well developed a ranked prioritization scheme is, the more prepared a nation is to deal with resource scarcity; however, the use of tiers may have both positive and negative consequences in view of the uncertainty associated with the actual characteristics of an emerging pandemic. While prior discussion of groups and their relative importance can lead to greater public acceptance at the time of a pandemic, the effect of reordering groups to reflect new evidence (during a crisis) is unclear. Emergency planners have further cautioned against planning in great detail in the face of uncertainty [14], an uncertainty evident in the wide variability in the definition of “high-risk” individuals. Nations should assess the risk-communication implications of exhaustively detailed prioritization.

Nations were urged by WHO to estimate the impact of a pandemic [5], yet only 40%—nearly all of which were developed nations—documented probable cases and/or deaths. We interpreted this to mean that priority setting is not consistently based on local conditions, or realities, especially in the case of the developing world. Nations should be encouraged and supported in priority setting based on individualized modeling or impact estimates.

There was some notable variation in the prioritization of key government decision makers, such as politicians. Of the nations that prioritized vaccine, 40% included key decision makers, and several more nations prioritized this group within the essential services category. Interestingly, when key decision makers appeared in rankings, this group ranked near the top of the list for vaccine (Tiers 1 and 3), but at the bottom for antivirals (Tier 7).

### Unresolved issues.

Our study highlights several unresolved issues. First, variation in priority setting may or may not represent a problem to be corrected, and may reflect concrete variation in local conditions. If this is the case, nations may disregard further WHO guidance that is not country specific, in addition to evidence-based.

Second, nations should be clearer with respect to the different antiviral strategies of treatment and pre or postexposure prophylaxis. Seven of 22 nations that prioritized antivirals did not differentiate among these strategies in allocating resources. Most countries that did differentiate, however, preferred treatment.

Third, if an antiviral stockpile is donated to a developing nation for the purpose of curbing a global pandemic at its source or for a humanitarian purpose, it is unclear at this time whether prioritization would follow a national prioritization scheme or one dictated by the donating body. Nations should plan for the use of national and potentially donated resources if they are willing to yield some control to a donating body.

Finally, it is unclear why vaccine and antiviral medications have been singled out for prioritization when many critical resources are likely to be scarce. Further guidance should address whether additional schemes for ventilators, N95 masks, and hospital beds are necessary, and should explore the special considerations involved in their distribution.

### Limitations of our analysis.

Our study had several limitations. First, we were restricted to nonprobability sampling because it was unclear how many countries have in fact developed written pandemic influenza plans. Our search strategy may be less effective at capturing the plans of developing nations, which may not publish plans on the Internet. Second, pandemic influenza plans are dynamic documents in various stages of revision. Any analysis that takes a snapshot of the state of planning has a certain shelf-life. Third, it is unclear how well any description of the level of planning detail actually captures the concept of preparedness; many of the plans noted that prioritization schemes are subject to change based on emerging epidemiology and social conditions, thus emphasizing the fact that flexibility is indispensable to preparedness.

Finally, because of variation in plan format, use of language, foreign-language translation, and the subjective nature of any categorization of diverse elements, our ability to represent the intent of planners is limited. Because of limited resources, we were unable to contact planners in each country and verify findings. In theory, however, the plans should stand as self-explanatory documents that clearly state pandemic policy.

## Conclusion

Attention to prioritization and its ethical implications may help to reduce death and disease burden, and minimize political destabilization and claims of injustice. Comparative analyses of national pandemic influenza plans provide a lens to reveal previously underaddressed considerations that can play a significant role in international preparedness against this global infectious disease threat.

## Footnotes

^a^ Plan provided by National Influenza Centers or personal contacts.

## Abbreviation

WHO: World Health Organization
